# HIV relies on neddylation for ubiquitin ligase-mediated functions

**DOI:** 10.1186/1742-4690-10-138

**Published:** 2013-11-18

**Authors:** Michael D Nekorchuk, Hamayun J Sharifi, Andrea KM Furuya, Robert Jellinger, Carlos MC de Noronha

**Affiliations:** 1 Center for Immunology and Microbial Disease, Albany Medical College, 43 New Scotland Avenue, Albany, NY 12208, USA; 2 Albany Medical Center Division of HIV Medicine, Albany Medical Center, 43 New Scotland Avenue, Albany, NY 12208, USA

**Keywords:** HIV, SAMHD1, APOBEC3G, Vif, Vpr, Vpx, UNG2, CUL4A, CUL5, NEDD8, MLN4924

## Abstract

**Background:**

HIV and SIV defeat antiviral proteins by usurping Cullin-RING E3 ubiquitin ligases (CRLs) and likely influence other cellular processes through these as well. HIV-2 viral protein X (Vpx) engages the cullin4-containing CRL4 complex to deplete the antiviral protein SAMHD1. Vif expressed by HIV-1 and HIV-2 taps a cullin5 ubiquitin ligase complex to mark the antiviral protein APOBEC3G for destruction. Viral Protein R of HIV-1 (Vpr) assembles with the CRL4 ubiquitin ligase complex to deplete uracil-N-glycosylase2 (UNG2). Covalent attachment of the ubiquitin-like protein side-chain NEDD8 functionally activates cullins which are common to all of these processes.

**Results:**

The requirement for neddylation in HIV-1 and HIV-2 infectivity was tested in the presence of APOBEC3G and SAMHD1 respectively. Further the need for neddylation in HIV-1 Vpr-mediated depletion of UNG2 was probed. Treatment with MLN4924, an adenosine sulfamate analog which hinders the NEDD8 activating enzyme NAE1, blocked neddylation of cullin4A (CUL4A). The inhibitor hindered HIV-1 infection in the presence of APOBEC3G, even when Vif was expressed, and it stopped HIV-2 infection in the presence of SAMHD1 and Vpx. Consistent with these findings, MLN4924 prevented Vpx-mediated depletion of SAMHD1 in macrophages infected with Vpx-expressing HIV-2, as well as HIV-1 Vif-mediated destruction of APOBEC3G. It also stemmed Vpr-mediated UNG2 elimination from cells infected with HIV-1.

**Conclusions:**

Neddylation plays an important role in HIV-1 and HIV-2 infection. This observation is consistent with the essential parts that cullin-based ubiquitin ligases play in overcoming cellular anti-viral defenses.

## Background

Retroviruses and their hosts carry evidence of enduring conflicts. Where hosts evolved antiviral restrictions, viruses developed countermeasures. Stalemates are reached and broken as balances between host and virus fall into disequilibrium. The antiviral restrictions have been found to act through various distinct mechanisms to block the infecting virus or to cripple its progeny. HIV, through specialized proteins, can commandeer cellular ubiquitin ligases to neutralize these blocks to infection.

HIV and SIV infections are restricted in macrophages, dendritic cells and resting CD4^+^ T-cells by SAMHD1, a dGTP-regulated deoxynucleotide phosphohydrolase (reviewed in [1]). SAMHD1 acts indirectly on incoming virus by depleting the nucleotide pool that is required for reverse transcription. HIV-2 and some simian immunodeficiency viruses (SIV) express Vpx to recruit SAMHD1 to the CRL4 complex and thereby trigger its ubiquitination and subsequent proteasomal degradation [2,3]. Of note, Pertel *et al.* identified another restriction distinct from SAMHD1, but also countered by Vpx [4]. DCAF1 is dispensable for this function. It's not clear however whether the remainder of the CRL4 complex is required for the viral defense [4].

HIV-specific restrictions, in addition to blocking virus on its way into cells can also sabotage virus during production. Members of the APOBEC protein family can diminish the infectivity of viruses produced in their presence [5]. APOBEC3G, a cytidine deaminase, targets residues in nascent negative strands during reverse transcription, resulting in the accumulation of G to A transitions in viral coding sequences. APOBEC3G can also physically block reverse transcription [6,7]. The anti-HIV activity of APOBEC3G requires its incorporation into virions during virus production. APOBEC3G is defeated by the viral Vif protein which acts as an adaptor between it and the cellular CUL5-Elongin B/C ubiquitin ligase complex [8]. This promotes the polyubiquitination and subsequent proteasomal destruction of APOBEC3G and thus its clearance from virus producer cells [8,9].

The viral defenses against innate host restrictions discussed here have two common features. First, all rely on host ubiquitin ligases. Second, the ubiquitin ligases engaged by these viruses are all thought to rely on neddylation to activate their function [10].

Neddylation is the addition of NEDD8, an 81–amino acid protein side-chain that is 60% identical and 80% homologous to ubiquitin, to a protein [11,12]. NEDD8, like ubiquitin, is ligated to lysine residues. This modification activates cullin ubiquitin ligase function by altering the conformation of the cullin protein [10].

The neddylation pathway is similar to that of ubiquitination. NEDD8 is activated by the neddylation activating enzyme 1 (NAE1) through an ATP dependent process. This results in the formation of NEDD8-AMP which is then linked with NAE1 through a thioester bond (reviewed in Brownell *et al.*[13]). NEDD8 is subsequently transferred to one of two E2 enzymes, UBC12 or UBE2F. These enzymes dock with an E3 ligase complex, which provides additional substrate specificity. UBC12 neddylates CUL1 through 4, while UBE2F neddylates CUL5 [14].

Here, we test the impact of neddylation on HIV infection and on HIV functions mediated through cullin-dependent ubiquitin ligases. In this work we inhibited neddylation using either MLN4924 [15] or a dominant negative form of the NEDD8-conjugating enzyme UBC12 (UBE2M). MLN4924 is an adenosine sulfamate derivative that forms an inhibitory NEDD8-AMP mimetic [13]. The dominant negative UBC12, with a C111S mutation, becomes stably conjugated to- and thereby sequesters NEDD8 before it can be transferred to CUL4 [16]. All cullin-containing ubiquitin ligases appear to rely on neddylation for efficient function. In this work we employed specific scenarios to link the requirement for neddylation with specific HIV functions.

We hypothesized that without efficient neddylation of cullin ubiquitin ligases, HIV-1 infection would be blocked in the presence of APOBEC3G. We further hypothesized that HIV-2 infection of macrophages would be severely hindered because Vpx would no longer trigger degradation of SAMHD1 to rescue reverse transcription. Finally we posited that HIV-1 Vpr would not trigger UNG2 degradation or other CRL4-dependent functions. The multi-faceted reliance of HIV on cullin-based ubiquitin ligases suggests that neddylation could be a strong therapeutic target.

## Results

### The neddylation inhibitor MLN4924 blocks HIV-2 infection of primary human monocyte-derived macrophages

HIV-1 Vpr and HIV-2/SIV Vpr and Vpx all engage CRL4 complexes [17-20]. Vpx from HIV-2 and SIV boosts infection of primary myeloid-derived cells [18-21], and has been shown to act through the CRL4 complex in this capacity [18-20]. We therefore hypothesized, that HIV-2 infection of macrophages would be inhibited in the presence of MLN4924 if neddylation is important for CRL4 function in this capacity. We based this on previous observations that Vpx triggers depletion of SAMHD1 through CRL4 [2,3,21] and that neddylation activates cullin-containing ubiquitin ligase complexes (reviewed in [10]). Vpx could alternatively use the complex without activation. Further, Hrecka *et al.*[22] showed that HIV-1 Vpr causes a boost in CUL4A neddylation. We thus considered the possibility that Vpx increases neddylation in an NAE1-independent mechanism that’s resistant to MLN4924.

To test whether Vpx-facilitated macrophage infection can be blocked by MLN4924, we infected primary human monocyte-derived macrophages (MDMs) with HIV-2 or HIV-2 with a frame-shift mutation in Vpx, Vpr or both. All of the viruses used were VSV-G-pseudotyped and expressed GFP in place of Nef, allowing infected cells to be identified using flow cytometry. One culture in each infection type was treated with MLN4924 and the other was exposed to the same conditions in the absence of the drug.

**Figure 1:**
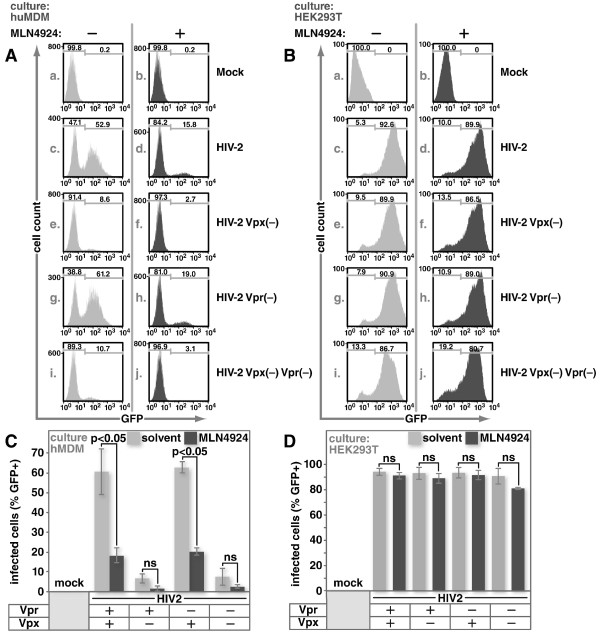
**MLN4924 inhibits infection of macrophages by HIV-2.** Primary human MDMs **(A)** or HEK293T cells **(B)** were pre-treated with 1 μM MLN4924 for three hours, and infected at an equivalent MOI with VSV-G-pseudotyped HIV-2 or HIV-2 with a frame-shift mutation in Vpx, Vpr or both as indicated. Panels **C** and **D** summarize data from multiple experiments and error bars show standard error. All viral constructs expressed GFP in place of Nef. GFP expression was detected using flow cytometry and used as an indicator of infection. The two-tailed Student’s t-test was used to determine whether differences between pairs of conditions are statistically significant. ns indicates comparisons where the differences were not significant at a level of 0.05.

As expected from previously published data, we observed robust infection with the viruses expressing Vpx and comparatively modest infection with the viruses lacking the capacity to express Vpx (Figure 1A, c and g versus e and i, and C). Addition of the neddylation inhibitor reduced infection of Vpx-expressing viruses to levels comparable with those lacking the capacity to express Vpx (Figure 1A, c and d, g and h, and C). While infection levels with Vpx(−) virus were relatively low, they were further reduced by MLN4924-treatment (Figure 1A, e and f, i and j, and C). It’s possible that the additional reduction in Vpx(−) virus infection is due to another requirement for cullin-containing ubiquitin ligases or due to an off-target effect on MDMs at this dose. If the MLN4924-mediated block of infection is due to the anti-viral activity of SAMHD1, we hypothesized that an equal dose of this compound would not block HIV-2 infection in HEK293T cultures. SAMHD1 does not exhibit anti-viral activity in this cell-type regardless of whether Vpr or Vpx is absent (Figure 1B, c and g versus e and i, and D). Addition of MLN4924 did not alter the overall pattern of infection in any of the cultures (Figure 1B left column versus right column). Slightly fewer cells were observed to be infected in the presence of the drug but this did not correlate with the presence of Vpx. Of note, we will show in subsequent figures that MLN4924 is biologically active in HEK293T cells at the dose used here and that the dose of MLN4924 used in these experiments has no significant impact on cell health during the time-frame of the experiments (Additional file 1: Figure S1).

### Neddylation is required for Vpx-mediated depletion of SAMHD1

**Figure 2:**
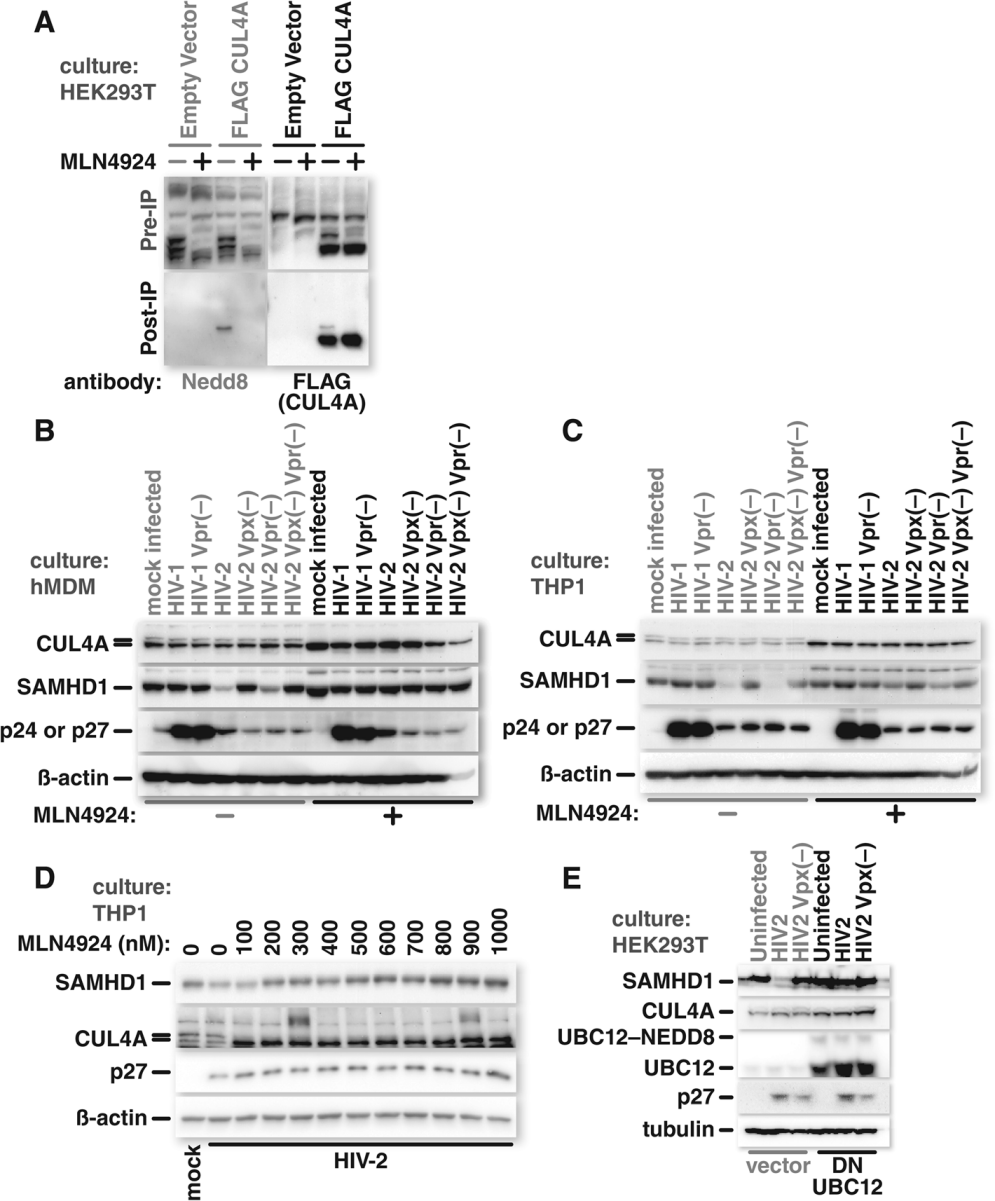
**Inhibition of neddylation blocks Vpx mediated depletion of SAMHD1.** HEK293T cells were transfected with an expression vector for FLAG–CUL4A or empty expression vector (pcDNA3.1(−)), and either treated with 250 nM MLN4924 or mock treated for four hours before harvest. Proteins were isolated from the cell lysates using beads coated with FLAG-specific antibody, and immunoblotted for either NEDD8 or the FLAG epitope as indicated **(A)**. Monocyte derived macrophages **(B)** or PMA-differentiated THP1 cells **(C)** were treated with 1 μM MLN4924 or mock treated and infected with VSV-G-pseudotyped HIV-1, HIV-1 with a frame shift mutation in Vpr, HIV-2 or HIV-2 with a frame-shift mutation in Vpx, Vpr or both. Cells were harvested and the lysates immunoblotted for CUL4A, β-actin, SAMHD1, and HIV p24/p27. PMA differentiated THP1 cells were mock treated or treated with increasing concentrations of MLN4924 before infection with HIV-2. Cells were harvested and the lysates were analyzed by western blotting as indicated **(D)**. HEK293T cells were transfected with either an empty expression vector (vector) or one for expression of dominant negative (DN) UBC12 and infected with VSV-G-pseudotyped HIV-2 or HIV-2 with a frame-shift mutation in Vpx. Cells were harvested and immunoblotted for CUL4A, tubulin, SAMHD1, UBC12 and HIV p24/p27 **(E)**.

We hypothesized that fewer macrophages were infected in the presence of the neddylation inhibitor MLN4924 because HIV-2/SIV requires the function of the CRL4 ubiquitin ligase to deplete SAMHD1. Vif should not play a role in the infections tested because the virus was produced in HEK293T cells which are devoid of functional APOBEC3G [9]. Since Vpx acts through the CRL4 complex, we tested the impact of MLN4924 on the neddylation status of CUL4A (Figure 2A).

HEK293T cells were transfected with either empty vector (pcDNA3.1(−)) or with a FLAG–CUL4A expression construct. The cultures were then either mock- or MLN4924-treated for four hours. The cells were lysed and proteins were isolated with FLAG-specific antibody to purify exogenously-produced FLAG–CUL4A. Immunoblotting with NEDD8-specific antibody showed a band only in the isolates from the cultures transfected with FLAG–CUL4A expression vector that were not treated with MLN4924 (Figure 2A). This band co-migrated with the upper band of the doublet detected with the FLAG-specific antibody which detects exogenously produced FLAG–CUL4A. In the MLN4924 treated sample, no signal was detected with the NEDD8 antibody and only a single, faster-migrating band was detected with the FLAG-specific antiserum. These results confirm that the upper band of the doublet is the neddylated CUL4A species and that MLN4924 blocks CUL4A neddylation.

After establishing that MLN4924 interferes with neddylation of CUL4A, we determined whether the compound blocks Vpx-mediated SAMHD1 depletion. Primary human monocyte-derived macrophages or PMA-differentiated THP1 cells were either mock- or MLN4924-treated. The cultures were then mock-infected or infected with HIV-1, Vpr-deficient HIV-1, HIV-2, or HIV-2 that cannot express Vpx, Vpr or both (Figure 2 panels B and C). Both cell types, when treated with the inhibitor, showed the collapse of the neddylated form of CUL4A into a single band, indicating that the neddylated form of CUL4A was no longer present. In the absence of MLN4924, SAMHD1 was markedly depleted in samples infected with Vpx-encoding HIV-2. Treatment with MLN4924, however maintained SAMHD1 levels despite infection with Vpx-encoding HIV-2. This occurred in both MDM and THP1 cultures. These data demonstrate that MLN4924 mediated inhibition of neddylation blocks HIV-2 Vpx-triggered SAMHD1 degradation.

In order to determine the minimal MLN4924 dose that’s required to block neddylation and Vpx-mediated SAMHD1 degradation in PMA-differentiated THP1 cells, we titrated the drug in 100 nM increments (Figure 2D). One hundred nanomolar MLN4924 was sufficient to cause a loss of the upper band that represents neddylated CUL4A. The loss of neddylation was accompanied by an intensification of the lower band. SAMHD1 levels only increased in 200 nM or greater concentrations of MLN4924, suggesting that there may be residual neddylation, and activity, at 100 nM that we did not readily detect by western blotting.

Dominant negative (DN) NEDD8 E2 ligase UBC12 provides an alternate means for blocking cullin neddylation [16]. Rather than blocking at the level of the E1 NEDD8 ligase, it sequesters NEDD8 at the level of the E2 ligase. Expression of dominant negative DN UBC12, like MLN4924, reduced the fraction of neddylated CUL4A in HEK293T cells (Figure 2E), albeit not as efficiently as the drug. The CUL4A bands shifted from a lighter doublet to a darker lower band in DN UBC12-expressing cells, much as with MLN4924 treatment. Further, the decrease in neddylated CUL4A was accompanied by a marked decrease in SAMHD1 depletion. HEK293T cells were used for these experiments because they are readily transfectable, unlike either THP1 cells or MDMs. Overall this data supports our observation that neddylation is important for Vpx-mediated SAMHD1-depletion regardless of whether it’s blocked with DN UBC12 or with MLN4924.

### Vif-induced depletion of APOBEC3G and efficient infectivity in the presence of APOBEC3G both rely on neddylation

Recent work by Stanley *et al.* showed that blocking the neddylation cascade hinders Vif-mediated depletion of APOBEC3G and thus infectivity in the presence of this cellular antiviral defense [23]. Here we confirm and support those earlier findings using a different system and demonstrate that neddylation is required for efficient Vif-mediated elimination of APOBEC3G. Based on the previous work, we hypothesized that inhibition of neddylation would block Vif-mediated depletion of APOBEC3G in virus producing cells. Virus from cultures treated with MLN4924, regardless of whether the viral genome encodes Vif, would thus package APOBEC3G and fail to efficiently infect target cells.

**Figure 3:**
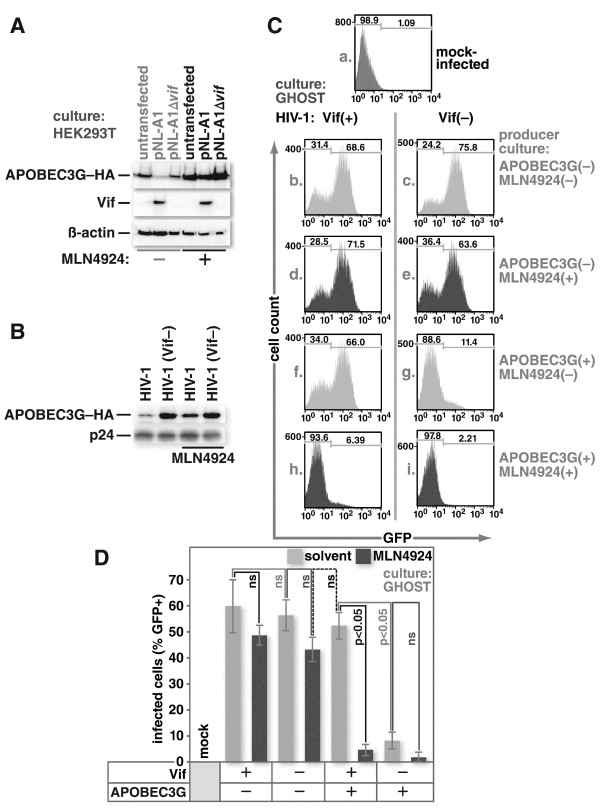
**MLN4924 inhibits Vif function.** HEK293T cells were transfected with an expression vector for APOBEC3G–HA, and either empty vector, pNL-A1, or pNL-A1Δvif. Twenty four hours later, the cells were either mock treated or treated overnight with 1 μM MLN4924. Cells were then harvested and immunoblotted for the HA epitope, β-actin, or HIV-1 p24 **(A)**. HEK293T cells were co-transfected with an expression vector for APOBEC3G-HA and either an expression vector for HIV-1 or one for a Δ vif HIV-1 along with an expression vector for the vesicular stomatitis virus glycoprotein (VSV-G). Twenty four hours later, cells were either mock treated or treated with 1 μM MLN4924, and cultured overnight. Virus-containing supernatants were collected and immunoblotted for HIV-1 p24 and the HA epitope **(B)**. HEK293T cells were co-transfected with HIV-1 or Δvif HIV-1 constructs along with an expression vector for VSV-G and either empty vector or APOBEC3G–HA. Twenty four hours later, cells were either mock treated or treated with 1 μM MLN4924, and left overnight. The virus-containing supernatants were collected and used to infect GHOST cells. Twenty four hours post infection; the cells were harvested, fixed, and analyzed for GFP expression by flow cytometry **(C)**. Panel **D** summarizes data from multiple experiments performed as indicated for panel **C** and error bars show standard error. The two-tailed Student’s t-test was used to determine whether differences between pairs of conditions are statistically significant. ns indicates comparisons where the differences were not significant at a level of 0.05.

To test this, HEK293T cells were transfected with an expression vector for C-terminal HA epitope-tagged APOBEC3G and either empty pcDNA3.1(−) expression vector, a Vif expression vector pNL-A1 or pNL-A1∆*vif* which is isogenic with pNL-A1 but does not express Vif. Expression of Vif, as expected, dramatically reduced the levels of APOBEC3G-HA (Figure 3A). Treatment with MLN4924 blocked Vif-mediated APOBEC3G depletion and allowed packaging of APOBEC3G into virions isolated from the culture supernatant (Figure 3B).

Infectivity, measured by GHOST cell assays, showed that virus produced in the presence of MLN4924, but in the absence of APOBEC3G, was unaffected whereas treatment with MLN4924 in the presence of APOBEC3G, resulted in a substantial reduction in infectivity (Figure 3C and D). Taken together, these data indicate that neddylation is required for Vif-mediated APOBEC3G degradation and that treatment of producer cells with MLN4924 results in APOBEC3G incorporation into virions and a subsequent loss of infectivity.

### HIV-1 Vpr-mediated degradation of UNG2 is neddylation-dependent

We and others have shown that UNG2 is a target for Vpr-directed degradation through the CRL4 complex [24,25]. Based on previous observations that neddylation activates cullin-dependent ubiquitin ligases, we hypothesized that this modification is required for efficient Vpr-mediated UNG2 degradation. If this is true, then a decrease in CUL4A neddylation should correlate with reduced Vpr-dependent protein depletion through CRL4. We thus tested whether MLN4924 blocks Vpr-dependent UNG2 depletion.

**Figure 4:**
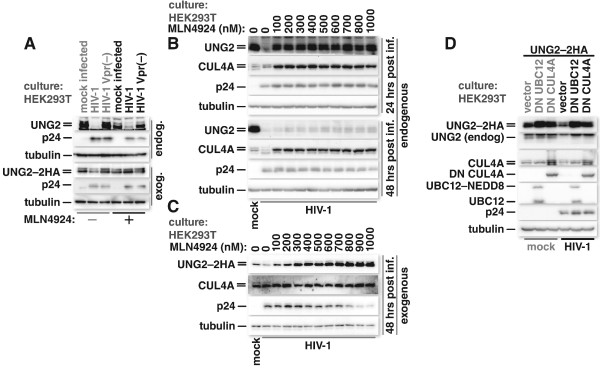
**Neddylation is important for Vpr mediated depletion of UNG2 through CRL4.** HEK293T cells were either mock treated or treated with 250 nM MLN4924 for four hours and infected with VSV-G-pseudotyped-HIV-1 or -HIV-1 with a frame shift mutation in Vpr. Twenty-four hours after infection, cells were harvested and the lysates immunoblotted for endogenous UNG2, HIV-1 p24 and tubulin (**A** endog.). HEK293T cells were transfected with an expression vector for UNG2 with two HA epitope tags (UNG2–2HA). Forty-eight hours later, cultures were mock treated or treated with 250 nM MLN4924 for four hours and then infected with VSV-G-pseudotyped-HIV-1 or -HIV-1 with a frame shift mutation in Vpr. Twenty-four hours later, cells were lysed and immunoblotted for the HA epitope, HIV-1 p24, and tubulin (**A** exog.). HEK293T cells were treated for 2 hours with concentrations of MLN4924 as indicated and then either mock infected or infected, in parallel, with a reduced MOI (approximately 2) of VSV-G-pseudotyped HIV-1. The cells were harvested for immunoblotting 24 and 48 hrs after infection. Blots were probed for endogenous UNG2, CUL4A, tubulin or HIV-1 p24 **(B)**. Twenty-four hours after transfection with UNG2–2HA expression vector, HEK293T cells were treated and infected as described for panel **B**. Forty-eight hours after infection, cultures were harvested for western blot analysis and probed for UNG2–2HA (HA), CUL4A, HIV-1 p24, or tubulin **(C)**. HEK293T cells were transfected with UNG2–2HA expression vector and either an expression vector (pcDNA3.1(−)) or ones for DN UBC12 or DN CUL4A. Twenty-four hours later cultures were mock infected or infected with VSV-G-pseudotyped-HIV-1. Twenty-four hours after infection cultures were lysed for immunoblotting and probed for UNG2–2HA (HA), endogenous UNG2, CUL4A, DN CUL4A (HA), DN UBC12, HIV-1 p24, or tubulin **(D)**.

Infection of HEK293T cells with Vpr-expressing HIV-1 caused a dramatic decrease in endogenous UNG2 levels (Figure 4A, endog.). Surprisingly, application of MLN4924 after infection barely blocked UNG2 depletion in these cultures. The observation that endogenous UNG2 depletion was not blocked more completely despite the robust loss of neddylated CUL4A species, led us to hypothesize that Vpr may be restricting UNG2 expression through both NEDD8-dependent and -independent mechanisms. The latter could be mediated through a cullin-independent ubiquitin ligase that is not impacted by a neddylation block, or by another mechanism altogether. Langevin *et al.*, for example, showed that Vpr expression hinders UNG2 production at the level of transcription [26]. Indeed, when we expressed UNG2, tagged with dual HA epitope tags (UNG2–2HA) from an expression vector using a CMV-IE promoter, UNG2–2HA levels were maintained upon infection with HIV-1 in the presence of MLN4924 (Figure 4A, exog.).

We tested the kinetics of endogenous UNG2 depletion, hypothesizing that Vpr coming in with the virion relies on CRL4-mediated UNG2 depletion while Vpr expressed from proviruses acts to silence transcription. We again used HEK293T cultures because we could look at both endogenous and exogenous UNG2. At 24 hours post infection we saw preservation of UNG2 with as little as 100 nM MLN4924, whereas at 48 hrs after infection we saw little preservation of UNG2 even at much higher concentrations of the drug (Figure 4B). We also tested exogenously expressed UNG2–2HA in this assay and found that while infection with Vpr-expressing HIV-1 caused UNG2—2HA depletion, MLN4924 treatment maintained UNG2—2HA at levels comparable to those in mock-infected cells in as little as 100 nM drug at 48 hrs post infection (Figure 4C). Interestingly, as we increased MLN4924 concentrations in cultures expressing exogenous UNG2–2HA we observed a decrease in p24 levels. This was not seen in cells expressing only endogenous UNG2 (Figure 4B). The decrease in p24 could reflect an anti-viral property of UNG2 when it’s over-expressed in the absence of either constitutive or Vpr-mediated turn-over.

We again used DN UBC12 to confirm that our observations did not rely solely on MLN4924. Here, we transfected HEK293T cells with expression vector for UNG2‒2HA either with empty vector, vector encoding DN UBC12 or vector encoding DN CUL4A (a truncated CUL4A that lacks the C-terminus, including the neddylation site). The cells were then either mock-infected or infected with HIV-1. Here both DN UBC12 and DN CUL4A boosted steady state UNG2 levels. This is consistent with our previous work showing that UNG2 is constitutively turned over through the CRL4 complex, albeit to a lesser degree than in the presence of Vpr [24]. Here both exogenous UNG2–2HA and endogenous UNG2 were depleted upon infection however only the depletion of the exogenous protein was blocked by DN CUL4A and DN UBC12 (Figure 4D).

**Figure 5:**
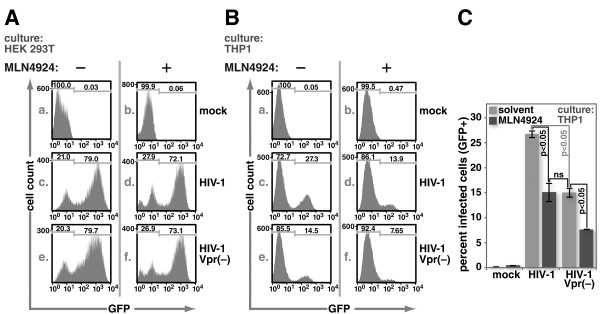
**MLN4924 inhibits HIV-1 infection of PMA-differentiated THP1 cells.** HEK293T cells **(A)** or PMA differentiated THP-1 cells **(B)** were pre-treated with 1 μM MLN4924 for three hours, and infected at an equivalent MOI with *env(−)*, VSV-G-pseudotyped-HIV-1 or -HIV-1 lacking Vpr. All viral constructs expressed GFP in place of Nef. Forty-eight hours post infection, cells were fixed, and infectivity was determined by enumerating GFP-expressing cells using flow cytometry. Panel **C** summarizes data from multiple replicates of experiments performed as indicated for panel **B**. Error bars show standard deviation. The two-tailed Student’s t-test was used to determine whether differences between pairs of conditions are statistically significant. ns indicates comparisons where the differences were not significant at a level of 0.05.

HIV-1 Vpr aids macrophage infection [27-29], however it does not deplete SAMHD1 in the context of an infection (Figure 2). We therefore tested whether inhibition of neddylation would interfere with HIV-1 infection of THP1 cells. We infected both HEK293T cells and PMA-differentiated THP1 cells with equivalent titers of VSV-G-pseudotyped *env*-deficient pNL4-3 that encoded (pNL4-3) or failed to encode Vpr (pNL4-3Δvpr) in the presence or absence of MLN4924. Vpr had no detectable impact on HEK293T infection (Figure 5A). Treatment with MLN4924, as in our other experiments, reduced infection slightly. Vpr caused a significant but modest increase in infection in PMA-differentiated THP1 cells, and this gain was neutralized in the presence of MLN4924 (Figure 5B and C). Interestingly, the infectivity of Vpr-deficient virus was reduced by about half in the presence of MLN4924 (Figure 5B and C).

## Discussion

Cullin4A and cullin5 are required for HIV to counteract at least two well-characterized antiviral factors, SAMHD1 and APOBEC3G. Neddylation is important for the function of ubiquitin ligases that rely on cullin proteins but the role of this modification is only now being tested in the context of HIV infections. Here we found that when neddylation was impaired either pharmacologically or by interference with the neddylation pathway though expression of a dominant negative UBC12, HIV-2 was no longer able to deplete SAMHD1. Inhibition of neddylation similarly blocked HIV-1 Vif-mediated reduction of APOBEC3G levels. As expected, interfering with Vpx and Vif function thus blocked HIV in contexts where these viral defenses were required for efficient infection. Neddylation thus plays a critical role in HIV biology and could present a target for antiviral intervention to block multiple viral functions simultaneously. The limitation to using a compound like MLN4924 therapeutically is however its potential for affecting all cullin-dependent ubiquitin ligases.

Vpx-mediated depletion of SAMHD1 showed a clear reliance on neddylation in primary human monocyte-derived macrophages, in differentiated THP1 cells and in HEK293T cells. Interestingly however, endogenous UNG2 levels were not well maintained when cultures were infected with Vpr-expressing HIV-1 in the presence of MLN4924. Vpr-mediated UNG2 depletion through CRL4 is a well-established phenotype [24,25,30]. Vpr assembles with both UNG2 and with the CRL4 complex and aids the assembly of UNG2 with the CRL4 complex [24,25]. While Vpr is not required for the depletion of UNG2 through CRL4, Vpr enhances this function [24]. Langevin *et al.* further showed that Vpr has a negative effect on the promoter of UNG2, but not on that of UNG1 [26]. Inhibition of neddylation should block constitutive and Vpr-mediated turnover of UNG2 like it blocked depletion of SAMHD1 and APOBEC3G, but shouldn’t interfere with Vpr-mediated inhibition of UNG2 transcription. Indeed, when we expressed UNG2—2HA from a CMV-IE promoter we observed depletion of this protein upon infection with Vpr-expressing virus but not after treatment with the neddylation inhibitor. It is likely that infection with a Vpr-expressing virus in the presence of MLN4924 thus interferes with endogenous UNG2 predominantly at the transcriptional level but not at the level of CRL4-directed depletion. These observations appear to separate the two mechanisms of UNG2 depletion by HIV-1 Vpr and show that both may be at play after an infection.

The role of UNG2 is not clear. Some work shows a negative impact of UNG2 on infection [30], other work shows a positive role [31-33], or no apparent effect [34,35]. The fact that HIV-1 Vpr employs two distinct mechanisms to reduce UNG2 levels suggests that UNG2 may be detrimental to the virus and establishes that Vpr, while being a relatively small protein, can block protein production at two points.

We did not observe a generalized Vpr expression-induced boost in the levels of CUL4 neddylation (Figure 2B and C) as might be expected based on the observations of Hrecka *et al.*[22]. This difference is not due to the use of primary hMDMs or THP1 cells because we saw the same level of neddylation in HEK293T cells infected with virus with or without Vpr (Additional file 2: Figure S2). The key differences between work shown here and that presented by Hrecka *et al.* is that we used infection to initiate Vpr expression rather than transfection of a Vpr expression vector and that we did not exogenously express components of the CRL4 complex. Our observation that UNG2 can assemble with CRL4 in the absence of Vpr [24] suggested that Vpr is boosting the efficacy of this interaction. The mechanism was unknown so increased neddylation was an attractive candidate. Our subsequent work however supports a model where Vpr is either recruiting or retaining more of the target proteins at the ubiquitin ligase [17].

HIV-1 Vpr also promotes G2 cell cycle arrest though interaction with the CRL4 ubiquitin ligase complex [17,22,36-39]. G2 cell cycle arrest is not linked to HIV-1 Vpr mediated UNG2 depletion and the ubiquitination target responsible for inducing arrest is not known [40]. While working to determine whether MLN4924 blocks Vpr-mediated G2 arrest we found that treating HEK293T cells with this agent blocked cells with a 4n complement of DNA suggesting that they were in either G2 or early mitosis (Additional file 3: Figure S3). This phenotype is likely because cullin-based ligases are required to maintain the delicate balance of cell cycle control proteins. This cell cycle block did not interfere with our analyses of protein depletion because cells under all infection conditions were treated with the drug prior to infection. Further, in our SAMHD1 experiments we worked predominantly with terminally differentiated and thus non-cycling cells.

Finally, recent work by de Silva *et al.* showed that Vpr enhances infection of monocyte derived dendritic cells with both single-round and replication competent virus [41]. This effect does not require DCAF1 and is thus thought to be independent of the CRL4 complex. This data is consistent with our THP1 infection data from Figure 5. The effects that we observed were relatively modest and will thus require additional experiments to determine whether CRL4 and other cullin-based ubiquitin ligases are dispensable for the Vpr-mediated infection boost.

## Conclusions

The work presented here demonstrates that neddylation is required for HIV to overcome at least two anti-viral restrictions and Vpr-mediated depletion of UNG2 through the CRL4 complex. The findings thus highlight the possibility of targeting neddylation for the development of therapeutic interventions against HIV infection.

Of note, while this manuscript was under revision Hofmann *et al.* published work showing that neddylation is important for Vpx-mediated depletion of SAMHD1 [42].

## Methods

### Ethics statement

Primary human monocytes were obtained from de-identified donors at the University of Nebraska Medical Center, Omaha, NE. The Albany Medical College Committee on Research Involving Human Subjects approved our protocol for the use of primary human monocytes and granted a category 4 exemption from consent procedures based on the anonymous nature of the samples.

### Cell lines

HEK293T and GHOST cells were maintained in Dulbecco’s Modified Eagle Medium (DMEM) with 10% fetal bovine serum, supplemented with 100 U/mL of penicillin, 100 μg/mL of streptomycin, and 2 mM L-glutamine.

Monocytic THP1 cells were differentiated using PMA treatment and cultured in RPMI 1640 with 10% fetal bovine serum, supplemented with 100 U/mL of penicillin, 100 μg/mL of streptomycin, and 2 mM L-glutamine.

Elutriated monocytes were obtained from de-identified donors (University of Nebraska Medical Center, Omaha, NE) and differentiated by treatment with recombinant human macrophage colony stimulating factor (rhM-CSF, Cell Sciences, Canton MA) in DMEM with 10% human serum for one week. Subsequently, the monocyte derived macrophages were cultured in DMEM supplemented with 10% human serum.

All cells were maintained at 5% CO_2_ at 37°C.

### Pharmacological inhibitor MLN4924

MLN4924 was a kind gift of Millennium Pharmaceuticals. The dry compound was dissolved in water at a concentration of 1 mM, and diluted as indicated.

### Plasmids and transfections

The pNL-A1 and pNL-A1-ΔVif plasmids were a kind gift from Dr. K. Strebel [43]. Molecular cloning of APOBEC3G‒HA was previously described in Stopak *et al.*[9]. The cloning of UNG2-2HA was previously described in Wen *et al.*[24]. The dominant negative UBC12 expression plasmid was a kind gift from Dr. T. Kamitani [16]. The FLAG‒CUL4A plasmid was a kind gift from Dr. J. Jin [44]. The Dominant negative CUL4A expression vector was described in Wen et al. [17].

Cells were transfected using the calcium phosphate method.

### Immunoprecipitations

HEK293T cells were transfected with either empty vector (pcDNA3.1(−)) or an expression vector encoding FLAG epitope-tagged cullin4A. Four hours before harvest, cells were treated with 250 nM MLN4924 or mock treated. Forty-eight hours after, cells were lysed in 0.5 mL ELB (50 mM HEPES, pH 7.3, 400 mM NaCl, 0.2% Nonidet P-40, 5 mM EDTA, 0.5 mM dithiothreitol, and Complete™ protease inhibitor mixture (Roche Applied Science, as per supplier’s instructions)) at 4°C for 30 min, then centrifuged to remove cellular debris. The supernatant was incubated with FLAG-specific M2 agarose resin overnight (Sigma-Aldrich). The resin-bound proteins were then washed three times with 1 ml of ELB buffer, and bound proteins were eluted using a 200 μg/mL solution of FLAG peptide (Sigma-Aldrich). The eluted proteins were immunoblotted for NEDD8 or for the FLAG epitope as indicated.

### Viral stocks and infections

The proviral clones used in the APOBEC3G experiment (pNL4-3*nef*(−)::HSA*env*(−),HIV-1; mouse HSA replaces Nef and pNL4-3*nef*(−)::HSA*env*(−) *vif* (−), mouse HSA replaces *nef*) were a kind gift of Dr. M. J. Lenardo [45].

The proviral clones used in the UNG2 depletion experiment (pNL4-3GFP*env*(−)*nef(−)* and pNL 4–3 GFP*env*(−) *vpr*(−)) were a kind gift of Dr. V. Plannelles [46].

The proviral clones used in the experiments with HIV-2 originated as a kind gift of Dr. M. Fujita, but have GFP in place of *nef* sequences upstream of the 3’ LTR. GFP encoding HIV-2 is based on pGL-AN, HIV-2Vpx(−) on pGL-St, HIV-2Vpr(−) on pGL-Ec, and HIV-2Vpx(−)Vpr(−) on pGL-St/Ec [47].

To generate viral stocks, the proviral clones were co-transfected with an expression construct for vesicular stomatitis virus G–protein (VSV-G) into HEK293T cells using the calcium phosphate method. The virus was harvested 48 hours after transfection.

### Immunoblotting

Harvested proteins were separated by SDS-PAGE, transferred onto a PVDF membrane (Millipore), and then probed with the indicated primary antibodies. The primary antibodies used were anti-FLAG: M2 (F1804, Sigma-Aldrich), anti-tubulin: (N-356 Amersham), anti-β actin (A5441, Sigma-Aldrich), anti-HA (12CA5, Roche), anti-UNG2 (a kind gift from Dr. Geir Slupphaug), anti-SAMHD1 (GTX83687, GeneTex), anti-CUL4A: (#2699S, Cell Signaling), and anti-UBC12: (#5641S, Cell Signaling). Please note that the UBC12 antibody also recognizes DN UBC12. The following reagents were obtained through the AIDS Research and Reference Reagent Program, Division of AIDS, NIAID, NIH: HIV-1 Vif Monoclonal Antibody (#319) (from Dr. Michael H. Malim, [48-50]), the p24 hybridoma supernatant (183-H12-5C) (from Drs. Bruce Chesebro and Hardy Chen [51]) and HIV-1 Vpr (1–50) Antiserum (from Dr. Jeffrey Kopp).

### Cell viability assay

PMA-differentiated THP-1 cells or HEK293T cells were either mock treated, treated with 1 μM MLN4924, or 5 μg/mL puromycin for 24 hours. Cell viability was assessed using the Cell Counting Kit 8 (Dojindo Molecular Technologies, Inc) according to the manufacturer’s protocol.

## Competing interest

The authors declared that they have no competing interest.

## Authors’ contributions

CN, MN, HS, and AF conceived of and designed the experiments. MN, HS, and RJ performed experiments. CN and MN wrote the manuscript. AF, HS and RJ revised the manuscript. All authors read and approved the final manuscript.

## Supplementary Material

Additional file 1: Figure S1 HEK293T and THP1 cultures treated with 1 μM MLN4924 exhibit metabolic activity similar to those of solvent-treated cultures. Cultures of PMA-differentiated THP1 or HEK293T cells were treated with solvent, 1 μM MLN4924 or 5 μg/mL puromycin for 24 hours. Cell viability was tested by measuring dehydrogenase activity as reflected by cleavage of WST-8 formazan reagent. Error bars represent +/− SE. Click here for file

Additional file 2: Figure S2 Vpr does not increase the neddylation of CUL4A. HEK293T cells were either mock treated or treated with 500 nM MLN4924 for 30 minutes and then infected with VSV-G-pseudotyped HIV-1 or HIV-1 with a frame shift mutation in Vpr. Twenty four hours after infection, cells were harvested and immunoblotted for CUL4A, tubulin, HIV-1 p24 or Vpr. Click here for file

Additional file 3: Figure S3 MLN4924 causes cell cycle arrest in HEK293T cells. HEK293T cells were either mock treated or treated with 500 nM MLN4924. Twenty-four hours post-treatment; the cells were harvested and the DNA was stained with propidium iodide. Cellular DNA content was assessed by flow cytometry. Click here for file
