# Supplementary figures and images for: HIV relies on neddylation for ubiquitin ligase-mediated functions
Source: Retrovirology. 2013 Nov 18;10:138. doi: 10.1186/1742-4690-10-138 (PMC3842660; doi:10.1186/1742-4690-10-138)

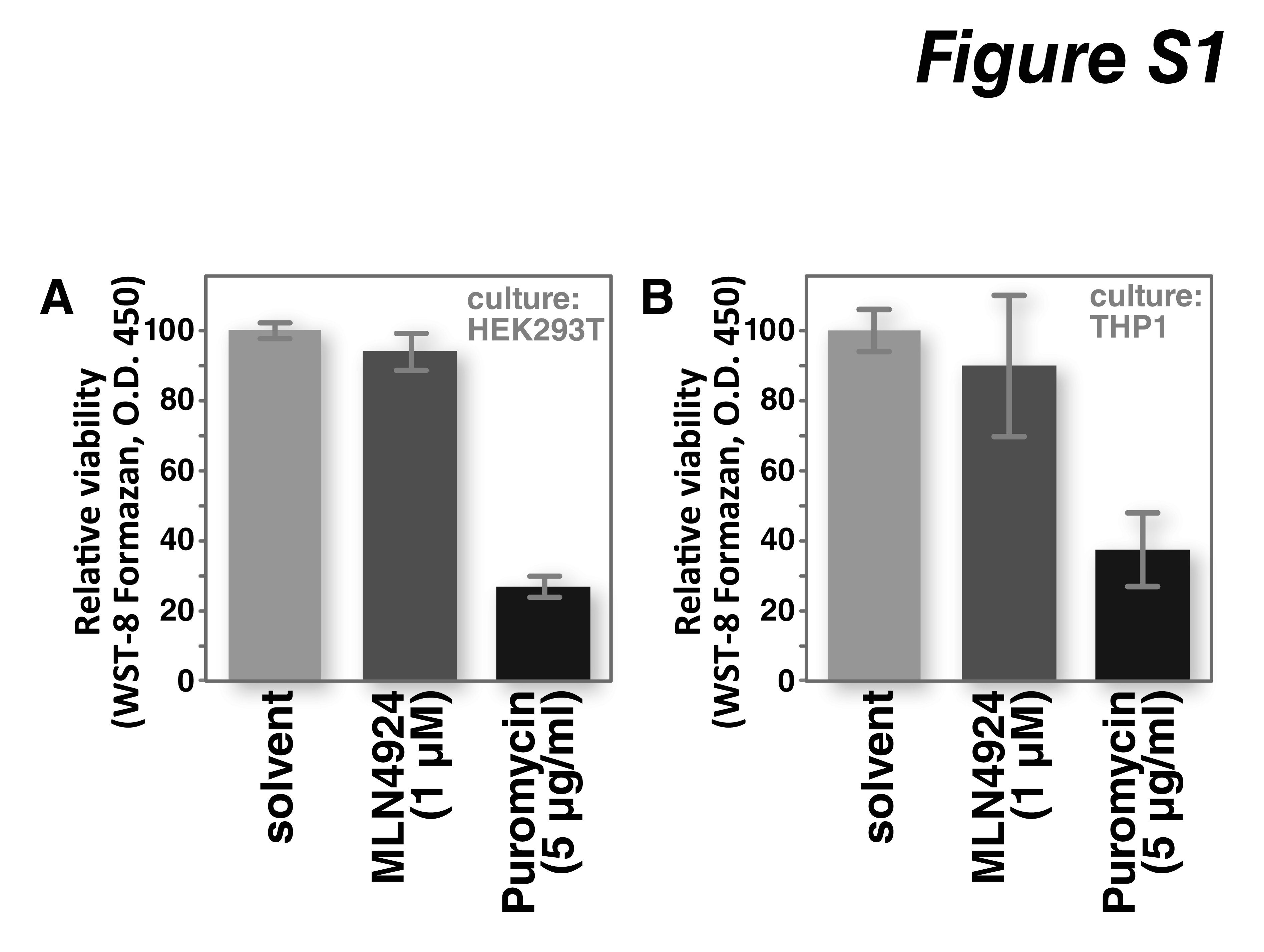

Supplement: Additional file 1: Figure S1 — HEK293T and THP1 cultures treated with 1 μM MLN4924 exhibit metabolic activity similar to those of solvent-treated cultures. Cultures of PMA-differentiated THP1 or HEK293T cells were treated with solvent, 1 μM MLN4924 or 5 μg/mL puromycin for 24 hours. Cell viability was tested by measuring dehydrogenase activity as reflected by cleavage of WST-8 formazan reagent. Error bars represent +/− SE. [file 1742-4690-10-138-S1.tiff]

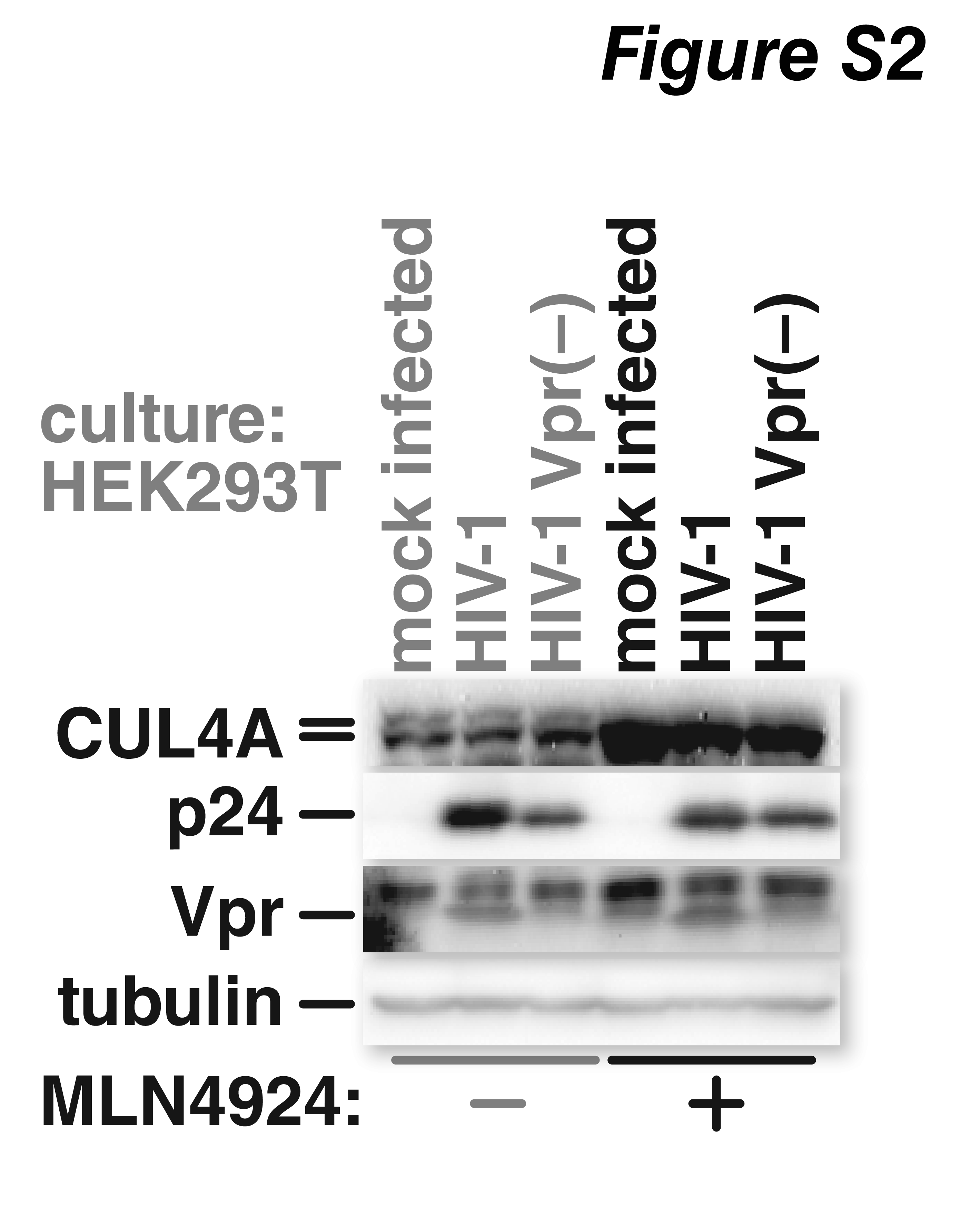

Supplement: Additional file 2: Figure S2 — Vpr does not increase the neddylation of CUL4A. HEK293T cells were either mock treated or treated with 500 nM MLN4924 for 30 minutes and then infected with VSV-G-pseudotyped HIV-1 or HIV-1 with a frame shift mutation in Vpr. Twenty four hours after infection, cells were harvested and immunoblotted for CUL4A, tubulin, HIV-1 p24 or Vpr. [file 1742-4690-10-138-S2.tiff]

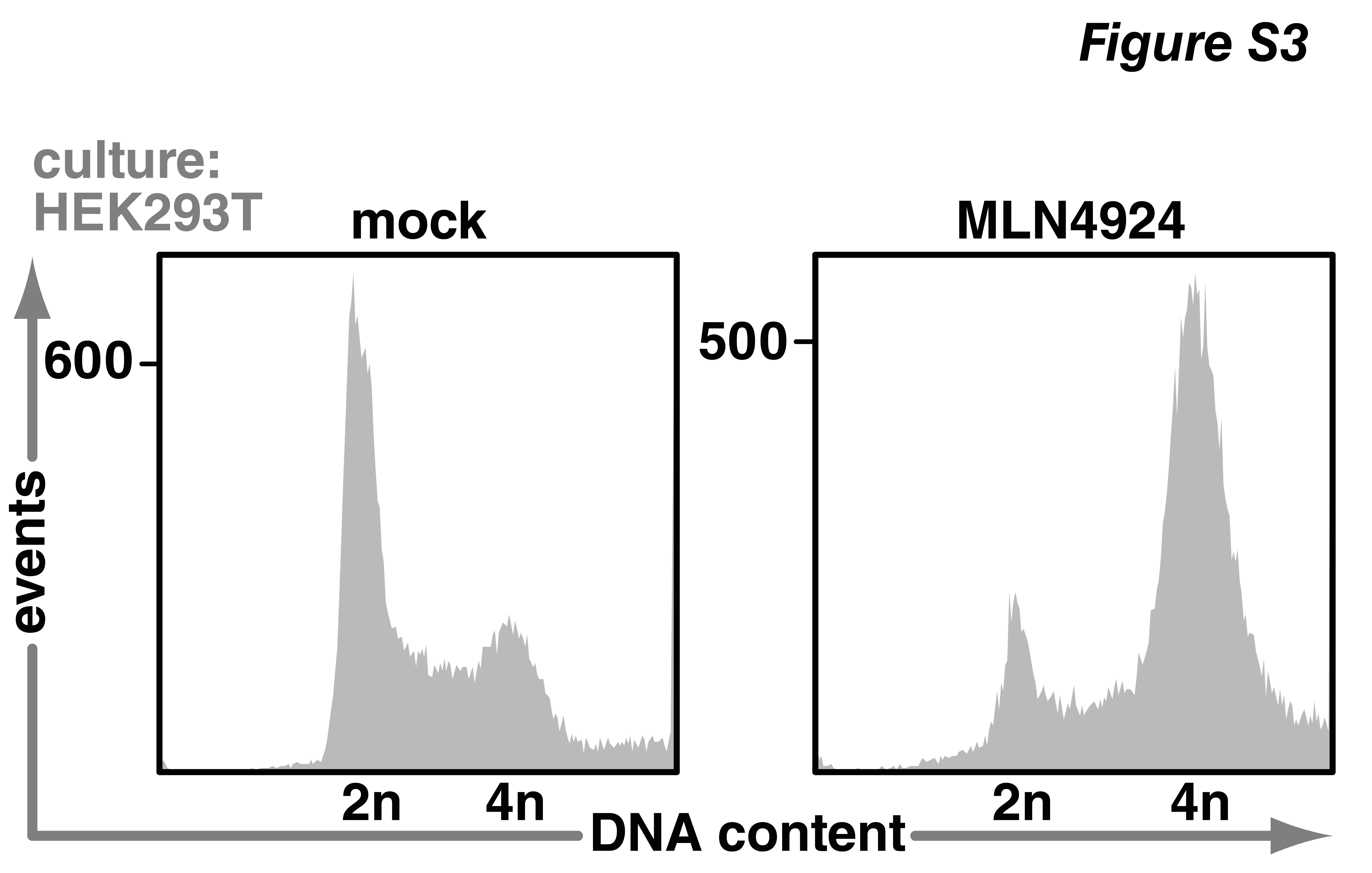

Supplement: Additional file 3: Figure S3 — MLN4924 causes cell cycle arrest in HEK293T cells. HEK293T cells were either mock treated or treated with 500 nM MLN4924. Twenty-four hours post-treatment; the cells were harvested and the DNA was stained with propidium iodide. Cellular DNA content was assessed by flow cytometry. [file 1742-4690-10-138-S3.tiff]
